# Diagnosis and preoperative predictive value of serum HE4 concentrations for optimal debulking in epithelial ovarian cancer

**DOI:** 10.3892/ol.2013.1339

**Published:** 2013-05-08

**Authors:** ZHIJUN YANG, ZHAOQIN LUO, BINGBING ZHAO, WEI ZHANG, JIEQING ZHANG, ZHUANG LI, LI LI

**Affiliations:** Department of Gynecologic Oncology, The Affiliated Tumor Hospital of Guangxi Medical University, Nanning, Guangxi 530021, P.R. China

**Keywords:** ovarian neoplasm, HE4, CA125, diagnosis, debulking

## Abstract

The aim of this study was to evaluate serum human epididymis protein 4 (HE4) concentrations for the diagnosis and preoperative prediction of optimal debulking in epithelial ovarian cancer. The concentrations of serum HE4 and CA125 in 180 epithelial ovarian cancer patients, 40 benign ovarian tumor patients and 40 healthy female subjects were determined using enzyme-linked immunosorbent assays (ELISAs). The value of determining the serum HE4 concentrations for the diagnosis and preoperative prediction of optimal debulking in epithelial ovarian cancer was also analyzed. The concentration of serum HE4 was 355.2±221.29 pmol/l in ovarian cancer, 43.86±20.87 pmol/l in benign ovarian tumors and 30.22±9.64 pmol/l in healthy individuals, respectively. The serum HE4 levels of patients with ovarian cancer were significantly higher compared with those in the other two groups (P<0.01), although there were no statistically significant differences (P>0.05) between the benign ovarian tumors and healthy individuals. The maximum diagnostic value was identified at an HE4 serum concentration of 67.52 pmol/l and the sensitivity and specificity were 84 and 96%, respectively. The area under the ROC curve was 0.944 (95% CI, 0.912–0.976; P<0.001) and the κ value of the diagnosis of epithelial ovarian cancer according to HE4 was 0.814 (P=0.000). The demarcation criterion was 600 pmol/l, where a value >600 mol/l indicates a lower possibility of optimal debulking. HE4 predicted that the sensitivity of the incomplete cytoreductive surgery was 77% and specificity was 32%. The concentration of serum HE4 is a useful marker for diagnosis and preoperative prediction for the ideal tumor cytoreductive surgery in epithelial ovarian cancer.

## Introduction

Ovarian cancer is the most lethal malignancy among females and the prognosis is poor since ovarian cancer is often at an advanced stage when it is detected (1–2). Early diagnosis of ovarian cancer is likely to improved the cure rate significantly. At present, the sensitivity and specificity of the clinically used markers of ovarian cancer are low for early diagnosis, so a number of studies have attempted to identify a more effective diagnostic marker (3). Human epididymis protein 4 (human epididymis gene product 4; HE4) is a marker of ovarian tumors which has significant potential for diagnosis (4). HE4 is a secreted protein coded by the gene WFDC2 and belongs to the lactic acid protein domain family (5–6). It has been demonstrated that HE4 mRNA is highly expressed in ovarian cancer tissue and not expressed in benign ovarian tissue (7). Moore *et al* (8) observed that HE4 was a useful single marker for differentiating between benign ovarian tumor and ovarian cancer patients. Köbel *et al* (9) analyzed the expression of a number of ovarian cancer markers in various pathological types of malignant ovarian tumors and observed high expression of HE4 in epithelial ovarian cancer. Since epithelial ovarian cancer accounts for 85–90% of ovarian cancer among the various pathological types, it is important to study the diagnostic value of HE4 for epithelial ovarian cancer. Cytoreductive surgery combined with platinum-based chemotherapy is the standard treatment for patients with ovarian cancer (10). Accurate preoperative assessments of the degree of malignancy and extent of metastasis are critical for optimal debulking, which is the best available approach for treating ovarian cancer at present (11). Previously, no tumor marker has been established to predict whether optimal debulking is likely to be achieved preoperatively. The aim of the present study was to appraise the diagnostic and preoperative predictive value of serum HE4 concentrations for optimal debulking in ovarian cancer.

## Patients and methods

### Source of specimens and clinical data

Serum specimens were obtained from ovarian neoplasm patients and diagnosed pathologically at the Department of Gynecologic Oncology of the Affiliated Tumor Hospital of Guangxi Medical University (Nanning, China). There were 180 malignant ovarian epithelial carcinoma patients, including 93 with ovarian serous adenocarcinoma, 38 with mucinous adenocarcinoma, 18 with endometrial adenocarcinoma, 14 with clear cell carcinoma and 17 with undifferentiated carcinoma. The median age was 37.6 years (range, 13–71 years). The surgical-pathological staging according the to FIGO (2004) staging criteria was 57 cases of stages II–II and 123 cases of stages III–IV. There were also 40 patients with benign ovarian tumors, including 13 with ovarian serous adenoma, 4 with benign ovarian teratoma, 10 with ovarian cysts and 13 with other types. The median age of the benign ovarian tumor patients was 43.8 years (range, 14–62 years). Additionally, 40 healthy female subjects were identified by physical examination, with a median age of 42 years (range, 33–50 years). The study was approved by the Ethics Committee of Guangxi Medical University. All patients received an explanation of the aims of the study, provided written informed consent and understood that they were able to withdraw from the study at any time without influencing their oncological or general medical treatment.

### Methods

#### Sample collection

Venous blood (3 ml) was obtained from each patient and placed in test tubes without anticoagulants. The blood samples were allowed to stand for 1 h at room temperature after specimen collection and the supernatant was collected after centrifuging at 3000 rpm. The samples were stored in a −80°C freezer until tested.

#### Determination of serum HE4

The concentrations of serum HE4 were determined using the double antibody sandwich enzyme-linked immunosorbent assay (ELISA) method. ELISA kits for serum HE4 detection were purchased from Fujirebio Diagnostics AB (Gothenburg, Sweden) and used according to manufacturer’s instructions.

#### Determination of serum CA125

Serum CA125 was detected using the electrochemiluminescent immunoassay (ECLIA) method. The ECLIA kit was provided by Roche Diagnostics (Mannheim, Germany) and the instrument used was a Roche El70 electrochemiluminescent analyzer which was used according to the manufacturer’s instructions. Serum CA125>35 U/ml was considered positive and serum CA125≤35 U/ml was considered negative.

#### Statistical analysis

Data were processed with SPSS 17.0 statistical software and the mean ± standard deviation was used to denote the measured data. The χ^2^ test was used to evaluate the enumeration data (the frequency of the positive or negative specimens). One-way analysis of variance was used to compare the concentrations of serum samples and the least significant difference two-sample t-test was used to compare pairwise mean values between groups. The specificity and sensitivity of the diagnosis of ovarian cancer using various HE4 concentrations were calculated using ROC curves and the concentration of HE4 with the greatest diagnostic value was selected as the best cut-off point. Diagnosis consistency was used to calculate κ values. The life table method was used to calculate survival rates and survival was compared with Kaplan-Meier survival curves and the log-rank test.

## Results

### Comparative analysis of the serum HE4 levels of each group

The concentration of serum HE4 was 355.2±221.29 pmol/l in ovarian cancer patients, 43.86±20.87 pmol/l in benign ovarian tumors and 30.22±9.64 pmol/l in healthy individuals. The difference between the HE4 serum levels of ovarian cancer patients and the other two groups was statistically significant (P=0.000) and the serum HE4 levels of ovarian cancer patients were significantly higher. The difference between the HE4 serum levels of the benign ovarian tumor lesion and healthy groups was not statistically significant (P>0.05). The results are shown in Table I.

### Analysis of the associations between serum HE4 levels, pathological types and clinical stages of ovarian cancer

The levels of serum HE4 were highest in the serous adenocarcinoma and clear cell carcinoma groups and the difference was statistically significant (P=0.019) compared with the other types of ovarian cancer. No statistically significant difference was observed between the mucinous adenocarcinoma, endometrial adenocarcinoma and undifferentiated carcinoma groups (P>0.05). In the comparison of the HE4 content between ovarian cancer stages I–II and III–IV, the difference was statistically significant (P=0.001). The results are shown in Table II.

### Diagnostic value of serum HE4 for ovarian cancer

#### ROC curve analysis

**Figure 1.**
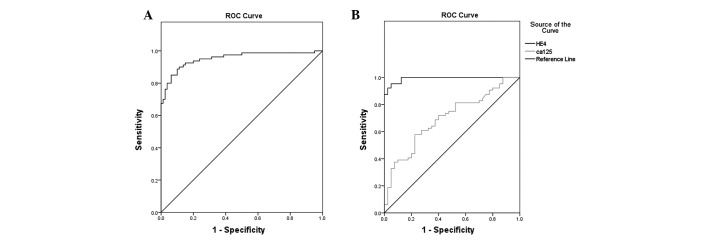
ROC curves of the diagnosis of (A) serum HE4 and (B) HE4 and CA125 for ovarian cancer. HE4, human epididymis protein 4.

An ROC curve was created which showed that the area under the curve was 0.984 (95% CI, 0.970–0.998, P<0.001) and the κ value was 0.814 (P=0.000). The maximum diagnostic value occurred when the cut-off for the diagnosis of serum HE4 for ovarian cancer was 65.52 pmol/l. The specificity and sensitivity were 96.2% and 83.8%%, respectively, and the positive predictive and negative predictive values were 95.7 and 85.6%, respectively. The results are shown in Fig. 1A.

#### Comparative analysis of diagnostic value between serum HE4 and CA125 for ovarian cancer

The specificity, sensitivity, positive predictive value and negative predictive value were all higher for HE4 diagnosis of ovarian cancer compared with CA125. The difference between the sensitivities was statistically significant (P=0.004). The difference in specificities was also statistically significant (P=0.003). The diagnostic performance of serum HE4 is superior to that of CA125, particularly for stage I–II patients. The difference between the sensitivities of the HE4 and CA125 of stage I–II patient groups was statistically significant (P=0.046). The results are shown in Table III.

#### Value of the combination of serum HE4 and CA125 in the diagnosis of ovarian cancer

Fig. 1B shows the ROC curves of serum HE4 and CA125 used alone or combination in the diagnosis of ovarian cancer. Table IV shows a comparison of the areas under the ROC curves. The diagnostic performance was compared between serum HE4, CA125 and HE4 + CA125, if HE4 and CA125 were positive. The specificity of HE4 + CA125 was significantly higher than HE4 or CA125 alone, while the sensitivity was lower compared with HE4 alone, but higher compared with CA125 alone. Table V shows the comparisons of sensitivity, specificity, positive predictive value, negative predictive value, positive likelihood ratio and negative likelihood ratio.

### Association of serum HE4 levels with the prognosis of ovarian cancer patients

All ovarian cancer patients were followed up until December 2011. The one-, two-, three- and four-year cumulative survival rates were 90, 62, 36 and 26%, respectively, and the median survival time was 28 months.

#### Kaplan-Meier survival curve analysis

**Figure 2.**
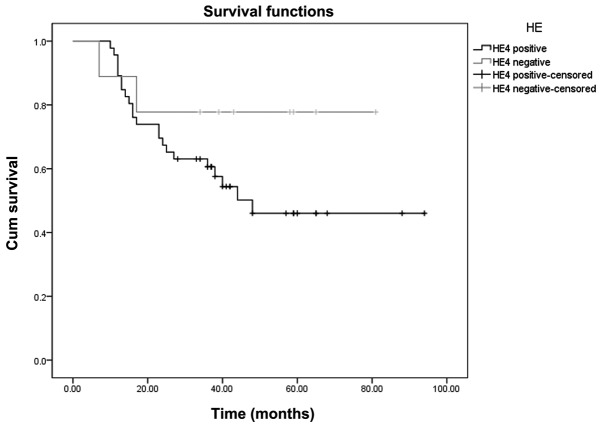
Kaplan-Meier survival curve of serum HE4-positive and negative ovarian cancer patients. HE4, human epididymis protein 4.

The Youden index of the ROC curve (Fig. 1A) is at the maximum, the concentration of the serum HE4 is 148.8 pmol/l. With 148.8 pmol/l as a positive threshold value, the Kaplan-Meier survival curves of serum HE4-positive and negative patients were compared. The log-rank test showed that the curves were significantly different (P=0.036). The results are shown in Fig. 2.

#### Cox model analysis of serum HE4 as an independent factor affecting the prognosis of ovarian cancer patients

The Cox proportional hazards regression model was analyzed in accordance with the following factors: serum HE4 >67.52 pmol/l, age, pathological type, clinical stage (I–II and III–IV), retroperitoneal lymph node metastasis, omentum metastasis, distant organ transfer and of postoperative residual focal factors. The results showed that the independent factors affecting the prognoses of ovarian cancer patients were the clinical stage, distant organ metastasis and postoperative residual tumors >2 cm. However, HE4, age, pathological type, retroperitoneal lymph node metastasis and omentum majus metastasis were not independent factors. The results are shown in Table VI.

### Association between the serum concentration of HE4 and CA125 and the possibility of optimal debulking in epithelial ovarian cancer

**Figure 3.**
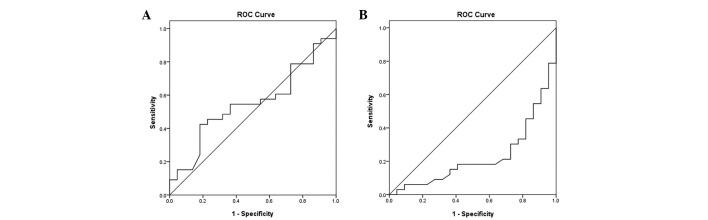
(A) ROC curve of the possibility of (A) serum CA125 and (B) serum HE4 for optimal debulking. HE4, human epididymis protein 4.

Debulking was performed in all patients. The results of debulking were compared with the preoperative serum concentrations of HE4 and CA125. With 500 U/ml as the demarcation criterion (CA125), the larger the numbers were, the lower the possibility of optimal cytoreduction surgery. CA125 predicted incomplete cytoreductive surgery with 72 and 30% sensitivity and specificity, respectively (Fig. 3A). The demarcation criterion of HE4 was 600 pmol/l, where a value >600 pmol/l indicates a lower possibility of the optimal cytoreduction surgery. HE4 predicted incomplete cytoreductive surgery with a sensitivity and specificity of 77 and 32%, respectively (Fig. 3B).

## Discussion

The present study showed that the concentration of HE4 in ovarian cancer patients was significantly higher than that in benign ovarian tumor and normal control patients (P<0.01), and no statistically significant differences were observed (P>0.05) between the benign ovarian tumor lesion and normal control groups. The mechanism of HE4 overexpression in ovarian cancer is not clear. However, the results of Berry *et al* (12) showed that the chromosomal region where HE4 is located is frequently amplified in breast cancer and ovarian cancer. However, few HE4 promoters are active in ovarian surface epithelial (OSE) cells, indicating that the increase in HE4 levels observed in ovarian cancer does not appear in normal ovarian epithelia culture. Moreover, HE4 is not expressed in the normal ovaries, early and late corpus luteum or fallopian tubes. Consequently, the level of serum HE4 may be used as marker for the diagnosis of ovarian cancer. The results of the present study are consistent with those of Moore *et al* (13) who detected the levels of serum HE4 in epithelial ovarian cancer (129 cases) and benign ovarian tumor patients (352 cases) and observed that HE4 was significantly increased in the epithelial ovarian cancer patients. The present study also was consistent with Köbel *et al*’s results (9). Kirchoff *et al* (6) observed that HE4 was expressed mainly in the distal epithelial cells of the epididymis and epithelial cells of the vas deferens. To further study the correlation between HE4 and ovarian cancer, Wang (14)*et al* studied the expression of HE4 in various ovarian tissues and revealed that HE4 was highly expressed in cancer tissue but not in normal ovarian tissue and pericancerous tissues. Another study showed that the HE4 secreted by ovarian cancer is a secreted protein resulting from N-glycosylation. Its molecular weight is less than CA125, so HE4 is more likely to be secreted into the blood than CA125 (15) and HE4 may be more effective than CA125 in early diagnosis. The present data also showed that the diagnostic value of HE4 was superior to that of CA125 in stage I–II patients. Montagnana *et al* (16) studied 46 ovarian cancer patients, 40 benign disease patients and a healthy control group and observed that the release of HE4 occurred earlier than CA125. The levels of HE4 had significantly increased in early ovarian cancer, while the levels of 40–50% CA125 did not increase. The present study also observed that the level of serum HE4 was the highest in serous carcinoma patients and the difference compared with other types of ovarian cancer was statistically significant (P<0.01). Drapkin *et al* (15) investigated the expression of HE4 in various types of ovarian cancer organization using an immunohistochemical method and observed that HE4 was expressed in 50% of ovarian clear cell carcinomas, 93% of ovarian serous ovarian cancer and 100% of endometrioid carcinomas of the ovary. However, it was not expressed in mucinous ovarian cancer and normal ovarian tissues. Therefore, the diagnostic value of HE4 may vary with the histopathological type. The present study also showed that the level of serum HE4 was highest in serous adenocarcinoma and clear cell carcinoma, compared with the other types of ovarian cancer, although no statistically significant difference was observed among mucinous adenocarcinoma, endometrial adenocarcinoma and undifferentiated carcinoma. Nolen *et al* (17) studied 65 tumor markers for the diagnosis of ovarian cancer and identified 34 significant markers. The diagnostic value of HE4 was the highest, followed by CYFRA 21–1, CA125 and CA-19–9. The sensitivity of the diagnosis of early ovarian cancer was improved from 74.2 to 91.7% by the combined detection of HE4 and CA125. It has been suggested that this combined detection is superior to the single detection of CA125 (18). Although the sensitivity of the joint detection of HE4 and CA125 is superior compared with the single detection of HE4, the difference is not significant and the specificity is lower compared with a single HE4 indicator. This may be associated with the false positive rate of the single detection of CA125. The present study showed that the sensitivity and specificity were 65 and 98.7%, respectively, for the combined detection of HE4 and CA125. This result was compared with HE4 used alone and it was observed that the sensitivity had decreased but the specificity was increased. When combining CA125 with HE4, if CA125 and HE4 were positive the combination was considered positive. The sensitivity and specificity of the combination (77.5% and 88.75%, respectively) were decreased compared with HE4 alone. Therefore, the present study suggests that the combined detection of HE4 and CA125 contributes to the differential diagnosis of benign or malignant pelvic masses, but is not superior to the single detection of HE4 for the early diagnosis of ovarian cancer.

Certain studies have investigated whether HE4 may be used as a marker to monitor disease progress and predict prognoses. The study of Xu *et al* (19) showed that the expression level of serum HE4 was significantly higher in a preoperative ovarian cancer group compared with healthy, benign ovarian epithelial tumor and borderline ovarian tumor groups and the differences were statistically significant (P<0.05). However, the serum HE4 expression level in postoperative ovarian cancer patients was significantly lower than the preoperative level, indicating that the level of serum HE4 may have play a role in the evaluation of surgical treatment. Although surgery is the major treatment option in ovarian cancer, its effect is often compromised by early and insidious extra-pelvic metastases, such as subphrenic and mesenteric root lesions, particularly in the superior abdomen region. The early diagnosis and accurate preoperative assessment of metastasis in ovarian carcinoma patients is critical for achieving optimal debulking and improving the five-year survival rate. The present study provides evidence that preoperative serous HE4 testing in the ovarian cancer patients may be regarded as an index for estimating the possibility of optimal cytoreductive surgery. The demarcation criterion was 600 pmol/l, where a value >600 mol/l indicates a lower possibility of optimal debulking by cytoreductive surgery. HE4 predicted that the sensitivity of incomplete cytoreductive surgery was 77% and the specificity was 32%. The present data also showed significant differences between the Kaplan-Meier survival curves of HE4-positive and negative patients (P=0.036, log-rank test). This suggested that the prognosis of ovarian cancer patients with higher concentrations of serum HE4 was worse than those without serum HE4. The Cox proportional hazards regression model was also used to analyze whether HE4 could be used as an independent prognostic factor, with the following factors: HE4-positive and negative, age, pathological type (epithelial and non-epithelial), stage (I–II and III–IV), distant organ transfer, ascites and postoperative residual focal factors. The results showed that the independent prognostic factors affecting the survival of ovarian cancer patients were clinical stage, distant organ metastasis and postoperative residual foci, while HE4, pathological type, lymph node metastasis and omentum majus metastasis were not independent prognostic factors affecting survival. This may be due to the small sample size and short follow-up period.

## Figures and Tables

**Table I.** Comparative analysis of the serum HE4 levels of each group (mean ± standard deviation).

Group	No. of cases	Content of HE4 (pmol/l)	P-value
Ovarian cancer	180	355.2±221.29	0.000^a^
Benign tumor	40	43.86±20.87	0.002^b^
Healthy control	40	30.22±9.64	0.453^c^

a Comparison of ovarian cancer with healthy control;

b comparison of ovarian cancer with benign tumor;

c comparison of benign tumor with healthy control. HE4, human epididymis protein 4.

**Table II.** Associations between serum HE4 levels, pathological types and clinical stages of ovarian cancer.

Clinicopathological factors	No. of cases	Content of HE4 (pmol/l)
Clinical stage		
I–II	57	226.43±196.87
III–IV	123	366.13±192.16
Pathological type		
Serous adenocarcinoma	93	448.11±159.59
Mucinous adenocarcinoma	38	299.90±206.27
Endometrial adenocarcinoma	18	309.90±206.27
Clear cell carcinoma	14	418.11±159.77

HE4, human epididymis protein 4.

**Table III.** Comparative analysis of diagnostic value of serum HE4 and CA125.

Group	HE4				CA125			
	Sensitivity (%)	Specificity (%)	Positive predictive value (%)	Negative predictive value (%)	Sensitivity (%)	Specificity (%)	Positive predictive value (%)	Negative predictive value (%)
Ovarian cancer	83.8	96.2	95.7	85.6	62.5	80.0	75.8	68.1
Stage I–II	70.4	96.2	86.4	90.6	44.4	80.0	60.0	68.1

HE4, human epididymis protein 4.

**Table IV.** Comparison of the area under the ROC curves of serum HE4 and CA125.

Detected marker	Area	Standard error	P-value	95% confidence interval
HE4	0.988	0.007	0.000	0.971–1.000
CA125	0.715	0.048	0.000	0.622–0.809

HE4, human epididymis protein 4.

**Table V.** Comparison of the diagnostic performance of serum HE4, CA125 and HE4 + CA125.

Marker	Sensitivity (%)	Specificity (%)	Positive predictive value (%)	Negative predictive value (%)	Positive likelihood ratio	Negative likelihood ratio
HE4	83.8	96.2	95.7	85.6	22.3	0.17
CA125	62.5	80.0	75.8	68.1	3.13	0.47
HE4 + CA125	65.0	98.7	98.1	73.8	52.0	0.35

HE4, human epididymis protein 4.

**Table VI.** Status of each factor in affecting the prognoses of ovarian cancer patients by Cox proportional hazards model analysis.

Factor	Regression coefficient	Standard error	Statistic	Degree of freedom	P-value	95.0% confidence interval	
						Lower limit	Upper limit
HE4	1.090	1.608	0.460	1	0.498	0.127	69.598
Age	0.028	0.019	2.146	1	0.143	0.991	1.067
Pathological type	−0.138	1.476	0.009	1	0.926	0.048	15.726
Stage	3.526	1.415	6.211	1	0.013	2.123	543.591
Lymph node	−0.328	0.544	0.365	1	0.546	0.248	2.090
Omentum majus	0.965	0.713	1.830	1	0.176	0.648	10.623
Ascites	0.491	0.544	0.815	1	0.367	0.562	4.748
Metastasis	−1.375	0.651	4.463	1	0.035	0.071	0.905
Postoperative residual foci	−2.758	1.064	6.721	1	0.010	0.008	0.510

HE4, human epididymis protein 4.
